# Real-Time Optical Detection of Isoleucine in Living Cells through a Genetically-Encoded Nanosensor

**DOI:** 10.3390/s20010146

**Published:** 2019-12-25

**Authors:** Shruti Singh, Maheshwar Prasad Sharma, Abdulaziz A. Alqarawi, Abeer Hashem, Elsayed Fathi Abd_Allah, Altaf Ahmad

**Affiliations:** 1 Department of Botany, School of Chemical and Life Sciences, Jamia Hamdard, New Delhi 110062, India; 189shrutis@gmail.com (S.S.); mpsharma@jamiahamdard.ac.in (M.P.S.); 2 Plant Production Department, College of Food and Agricultural Sciences, King Saud University, P.O. Box 2460, Riyadh 11451, Saudi Arabia; alqarawi@ksu.edu.sa (A.A.A.);; 3 Botany and Microbiology Department, College of Science, King Saud University, P.O. Box. 2460, Riyadh 11451, Saudi Arabia; habeer@ksu.edu.sa; 4 Mycology and Plant Disease Survey Department, plant pathology Research Institute, ARC, Gaza 12511, Egypt; 5 Department of Botany, Aligarh Muslim University, Aligarh 202002, India

**Keywords:** isoleucine, Förster/Fluorescence Resonance Energy Transfer, nanosensor, LivJ, periplasmic binding protein

## Abstract

Isoleucine is one of the branched chain amino acids that plays a major role in the energy metabolism of human beings and animals. However, detailed investigation of specific receptors for isoleucine has not been carried out because of the non-availability of a tool that can monitor the metabolic flux of this amino acid in live cells. This study presents a novel genetically-encoded nanosensor for real-time monitoring of isoleucine in living cells. This nanosensor was developed by sandwiching a periplasmic binding protein (LivJ) of *E. coli* between a fluorescent protein pair, ECFP (Enhanced Cyan Fluorescent Protein), and Venus. The sensor, named GEII (Genetically Encoded Isoleucine Indicator), was pH stable, isoleucine-specific, and had a binding affinity (K_d_) of 63 ± 6 μM. The GEII successfully performed real-time monitoring of isoleucine in bacterial and yeast cells, thereby, establishing its bio-compatibility in monitoring isoleucine in living cells. As a further enhancement, in silico random mutagenesis was carried out to identify a set of viable mutations, which were subsequently experimentally verified to create a library of affinity mutants with a significantly expanded operating range (96 nM–1493 μM). In addition to its applicability in understanding the underlying functions of receptors of isoleucine in metabolic regulation, the GEII can also be used for metabolic engineering of bacteria for enhanced production of isoleucine in animal feed industries.

## 1. Introduction

Isoleucine is an essential, aliphatic, branched-chain amino acid (BCAA) that serves as a host of physiological functions in the human body. Isoleucine is known to have a hypoglycemic effect in humans and has been found to inhibit the degeneration of muscle tissue by depressing hepatic gluconeogenesis [1]. It serves as one of the main nitrogen sources for alanine and glutamine synthesis in muscles. Besides glucose and protein metabolism, isoleucine promotes intestinal health through enhanced mucin production and amino acid transportation [2]. Isoleucine also affects brain function by influencing the transport of large neutral amino acids across the blood-brain barrier [3]. In the field of predictive diagnosis, isoleucine (among other BCAAs) has been postulated as a plausible bio-marker for major depression in human subjects. Isoleucine (along with leucine) can act as a predictive bio-marker of type 2 diabetes and insulin resistance [4,5]. Other diseases for which isoleucine can be used as a bio-marker, include Huntington’s disease and Maple Syrup Urine Disease. Furthermore, evidence shows that there are specific actions of food for the activation of intestinal receptors [6]. The activated receptors might be involved in the regulation of food intake, small intestine mobility, and neuron reflexes [7]. However, investigations on the specific receptors of isoleucine have not been carried out in detail. Knowledge of the receptors of isoleucine will be helpful in understanding its physiological roles. A tool that can measure the flux of isoleucine in living cell will expand our vision of the novel functions of isoleucine and its receptors in humans and animals.

In plant biology, isoleucine has been linked intricately with plant development. Partial isoleucine deficiency resulted in the impairment of root development in *Arabidopsis thaliana* [8]. Herbicides, that inhibit the bio-synthesis pathway of BCAAs, including isoleucine, have long been used for selective weed control. The oxidation of isoleucine feeds the mitochondrial electron transport chain in plants [9]. Isoleucine also has an important role in plant defense and stress by forming a bio-active signaling conjugate with jasmonic acid [10,11]. However, similar to isoleucine’s role in human and animal physiology, its role in plant physiology is not well-understood at the cellular and subcellular levels, due to the technical limitations in observing real-time measurements of isoleucine in single-cell resolutions.

In the industry, isoleucine has significant commercial importance as it is used as an important ingredient of feed in the poultry industry, pig-farming, and fish-farming. More recently, isoleucine has gained popularity as a nutritional supplement for muscle building. Owing to its increased demand and usages, the annual production of isoleucine has steadily increased from less than 400 tonnes in 1999 to more than 2000 tonnes today. Industrial biotechnology has revolutionized the synthesis process. The industrial production of isoleucine primarily relies on *E. coli* and *C. glutamicum* mutants that over-produce isoleucine, and avoiding byproduct formation in the fermentation process. Many approaches have been used for the identification of bacterial strains with high isoleucine yield and limited by-product formation [12]. Nevertheless, sincere efforts are being made in industrial biotechnology to enhance microbial production of isoleucine. This can be achieved by developing high isoleucine-producing bacterial strains through metabolic engineering. However, metabolic engineering approaches in producing elite bacterial strains requires the knowledge of the flux of a metabolite in metabolic network. To understand the metabolic flux, a highly-sensitive and non-invasive tool is a pre-requisite.

Methods, such as spectrophotometric assays [13], high performance liquid chromatography (HPLC) [14], radioisotopes [15], and mass-spectrometry [16] have been used in the past to measure isoleucine concentrations in plasma and bacterial cells. However, these techniques are highly invasive, have limited cellular resolution, and provide only a snapshot of the metabolite dynamics in cells. This prevents detailed studies to be undertaken, in order understand the isoleucine mechanism in living cells. To mitigate these limitations, a number of biosensors were developed for BCAAs. For example, Chino et al. reported a dye-based fluorescent sensor Gln149Cys-M for BCAAs [17]. Mustafi et al. developed a transcriptional regulator-based cellular biosensor for BCAAs [18]. However, both of these sensors respond to all branched-chain amino acids as a group and are only limited to in vitro/bacterial applications. Amongst sensors responding to specific BCAAs, Mohsin et al. constructed a Förster/Fluorescence Resonance Energy Transfer (FRET)-based sensor with a specific response to leucine for use in bacterial and yeast cells [19]. However, no reported instances of a biosensor exist that respond specifically to isoleucine and can be used in vivo.

As noted above, one of the physical phenomena that have been successfully exploited in measuring intra-cellular distances on the scale of nanometers is the FRET. This occurs when a donor fluorophore is in close proximity to an acceptor fluorophore, and emission spectrum of donor fluorophore overlaps with emission spectrum of acceptor fluorophore. Since FRET efficiency depends on the relative orientation and is inversely proportional to the sixth power of the donor-acceptor distance. This basic principle has been used to construct nanosensors for a number of metabolites like sugars, amino acids, and metal ions [20,21,22]. A periplasmic binding protein (PBP), from a bacterial source usually, serves as the scaffold for such a sensor, to which FRET pair of fluorescent proteins are attached. In the presence of its substrate, the PBP undergoes a reversible large conformational change via a hinge-bending motion resembling Venus flytrap, thereby, bringing the attached fluorophores closer. This, in turn, results in an increase in FRET efficiency. Hence, metabolite concentrations act as a transducer of the FRET ratio of the sensor affording extremely fine-grained measurements in space and time.

Apart from translating metabolite concentration into a fluorescence ratio readout, FRET-based sensors have other very practical desirable characteristics. These sensors are ratiometric, and unlike the previously reported intensity-based sensor for BCAAs [17], their measurements are unaffected by background auto-fluorescence, sensor protein concentrations, or excitation wavelength. Since the nanosensors are genetically encoded, they cannot only be targeted specifically to sub-cellular organelles, such as mitochondria, but also do not suffer from issues, such as cell toxicity that plague molecular dye-based sensors. All these factors make FRET-based nanosensors an attractive choice for visualizing metabolite fluxes in living cells.

Given the above, this study presents the designing and construction of a genetically encoded FRET-based nanosensor for monitoring of isoleucine in living cells in a non-invasive manner. To the best of our knowledge, the current study is the first application of FRET principle for the construction of an isoleucine specific sensor with demonstrated capability for live cell imaging in both prokaryotic and eukaryotic systems.

## 2. Materials and Methods

### 2.1. Molecular Modeling

The leucine/isoleucine/valine-binding protein (LIVBPs, also known as LivJ) from *E. coli* (strain K-12) was selected as the reporter element for the preparation of the isoleucine sensor. The crystal structure of LivJ in complex with isoleucine (PDB ID-1Z17) at a resolution of 1.96 Å was obtained from the RCSB PDB (The Research Collaboratory for Structural Bioinformatics Protein Data Bank). Docking studies were done using Schrödinger suite’s graphical user interface Maestro 11.4. The crystal structure of LivJ was prepared using the protein preparation wizard which has processing, modification, and refinement tools. It was used to assign bond orders, add missing hydrogens, and delete waters beyond 5 Å, whilst keeping the necessary waters intact. In the refinement section, H-bond assignment was used for optimizing the hydrogen bonding network at a specific pH. This is necessary as adjusting the pH changes the protonation states of residues and ligands, and is useful in accurately reflecting experimental conditions. Next, ligand preparation was done using Ligprep tool, in which different ionization states of ligand were generated at the working pH of ligands in the body. This was followed by generating the grid of optimum dimensions at the active site using receptor grid generation tool. Lastly, isoleucine was docked to LivJ using Glide ligand docking tool in extra precision (XP) mode taking the best suitable binding pose. To rationalize docking results, binding energy calculations were done using the Prime MMGBSA module.

### 2.2. Desiging and Construction of Nanosensor

**Figure 1:**
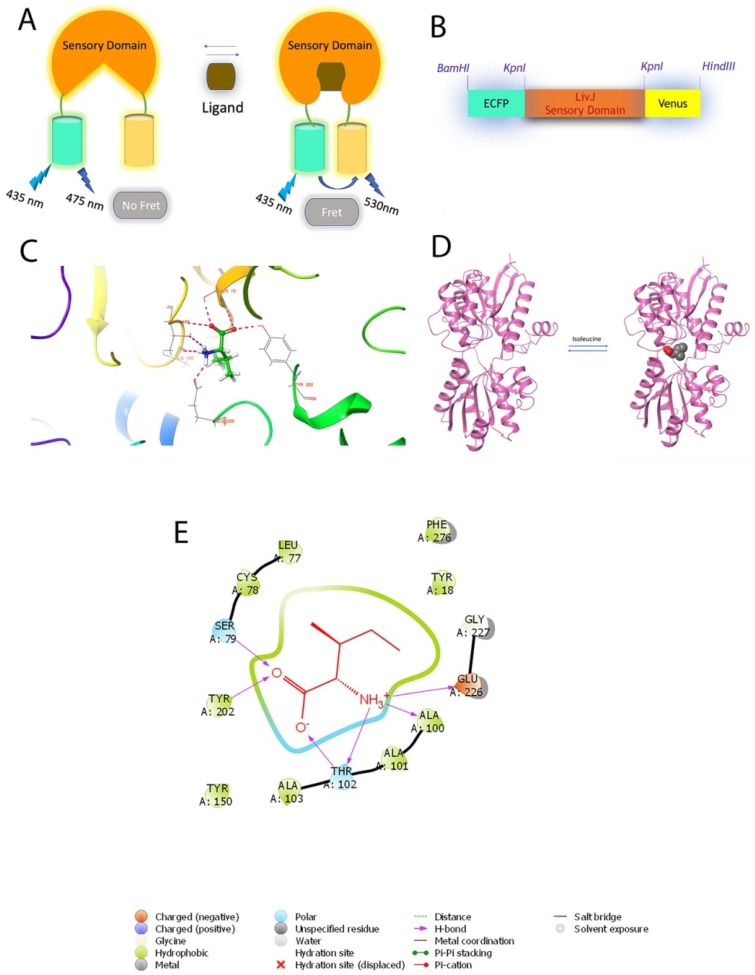
Designing of nanosensor. (**A**) Generic representation of the Förster/Fluorescence Resonance Energy Transfer (FRET) mechanism with sensor protein attached to Enhanced Cyan Fluorescent Protein (ECFP) and Venus. In the absence of ligands, the sensory domain exists in an open form with the inter-fluorophore distance being large enough so as to preclude any FRET (fluorescence emission only by ECFP at 475 nm). On binding with the ligand, the sensor takes on a closed form bringing the attached fluorescent proteins within FRET permissible range, resulting in fluorescence emission by Venus at 530 nm. (**B**) Schematic representation of the GEII sensor with LivJ as the sensory domain and ECFP/Venus as the fluorescent pair. Restriction sites are also depicted. (**C**) Docking pose of LivJ in complex with isoleucine. Ball and stick form represent isoleucine. (**D**) Pictorial representation of the open and closed forms of the LivJ binding protein in the absence, and presence of isoleucine, respectively. (**E**) Interactions of the active binding site in the LivJ-isoleucine bound complex as determined from docking studies carried out using Schrödinger suite.

The design of construction of nanosensor is given in Figure 1. The gene sequence of LivJ binding protein was retrieved from the NCBI. The LivJ gene without periplasmic leader sequence was amplified using genomic DNA of *E. coli* K-12. Gene-specific primers were designed: Forward primer was 5′-CGG**GGTACC**GAAGATATTAAAGTCGCGGTCG-3′ and reverse primer was 5′- CGG**GGTACC**ATCGGTCGCCGTGCCGTTG-3′. Bold sequence of the primers indicates *Kpn*I restriction site. The amplified LivJ gene was ligated with Enhanced Cyan Fluorescent Protein (ECFP) and Venus DNA sequences at 3′ and 5′, respectively. The resultant nanosensor, pRSET-B_ECFP-LivJ-Venus, was named as Genetically Encoded Isoleucine Indicator (GEII). Nucleotide sequencing of the complete GEII construct was carried out to verify the authenticity of the developed sensor (Figure S1).

The pRSET-B-ECFP-LivJ-Venus was transformed into the *E. coli* BL21 (DE3) strain through electroporation for sensor protein expression. The ECFP-LivJ-Venus construct was then shuttled into the yeast expression vector, pYES-DEST52. The resultant construct was transformed into *Saccharomyces cerevisiae*/URA3 strain and grown in YEPD medium at 30 °C with proper aeration.

### 2.3. Expression and Purification of the Nanosensor

Single colony of *E. coli* BL21DE3 containing pRSET-B-ECFP-LivJ-Venus was inoculated in Luria Bertini (LB) broth for 22 h at 21 °C. Induction of the culture was carried out using 1 mM isopropyl-d-1-thiogalactopyranoside. The culture was further grown for 36 h at 21 °C for the expression of the nanosensor. Thereafter, harvesting of bacterial cells was carried out through centrifugation at 6500× *g* for 20 min at 4 °C. The harvested bacterial cells were resuspended in 20 mM Tris-Cl (pH 8.0). After cell lysis through ultrasonication, centrifugation process was repeated, and the protein fraction was collected and loaded into a column. The purification of the nanosensor protein was carried out using Ni-NTA His-tag affinity chromatography. Elution of the nanosensor protein was done using 20 mM Tris-Cl and 100 mM imidazole (pH 8.0). The nanosensor protein was run on sodium dodecyl sulphate polyacrylamide gel electrophoresis (SDS-PAGE, 12% acrylamide) to check the purity of the protein (Figure S2).

### 2.4. Characterization of the Nanosensor

Fluorescence spectrophotometer (RF5301PC, Shimadzu, Europe) was used to obtain fluorescence emission spectra by exciting ECFP at 435 nm and recording emission intensities from 480 to 580 nm without adding isoleucine and after addition of 1 mM isoleucine. For the characterization process, 20 mM each of 3-(N-Morpholino) propanesulphonic acid (MOPS), Phosphate-buffered saline (PBS) and Tris-buffered saline (TBS) with pH ranging from 5.0 to 8.0 were prepared. Dilution of the purified sensor was done to reach the concentration of 0.20 mg/mL. Ratio of emission intensities of Venus/ECFP was recorded using microplate reader (DTX880, Beckman Coulter, USA).

Since MOPS buffer was found to be the most stable, the stability of diluted sensor protein was checked in the presence of 10 mM isoleucine, as well as in the absence of isoleucine using MOPS buffer (pH range: 5.0–8.0). While, in the acidic pH range, the sensor protein’s FRET ratio varied with the changing pH, it was found to be stable in the alkaline pH (7–7.5) range. Since, pH 7.5 typifies the physiological pH, it was chosen to perform further experiments.

Sensor specificity and functionality were checked with different amino acids, such as leucine, isoleucine, valine, methionine, arginine, cysteine, alanine, asparagine, aspartic acid, glutamine, glutamic acid, glycine, histidine, lysine, and serine at 0, 1 mM and 10 mM concentration. The FRET ratio analysis was performed in 96-well microplate reader using the excitation filter/slits (430/20 nm) and emission filters/slits for ECFP (485/20 nm), and Venus (535/25 nm) respectively. Furthermore, the sensitivity of sensor protein was tested against 10 mM of different metals ions present in the human body i.e., NaCl, CaCl_2_, MgCl_2_, and KCl.

To calculate the affinity of the GEII protein, ratio of emission intensities of Venus/ECFP was measured at different concentrations of isoleucine, ranging from nanomolar to millimolar to obtain the ligand titration curve. Binding constant (K_d_) was determined by fitting the ligand titration curve in a binding isotherm equation,

S = (r − R_min_)_/_(R_max_ − R_min_) = [S]_bound/_[P]_total_ = n[S]/(K_d_ + [S]) (1)

where S represents saturation, r is the FRET ratio, R_min_ and R_max_ denote the minimum, and maximum FRET ratio respectively, [S]_bound_ is concentration of the bound substrate, [P]_total_ is the total concentration of the protein, [S] is the concentration of the substrate, n is the number of binding sites, and K_d_ denotes the dissociation constant. Non-linear regression using Generalized Additive Model (GAM) with cubic regression splines in R software was used to obtain the best fit curve for ligand titration data.

### 2.5. Affinity Mutants

Affinity mutations were generated through point mutation using the Bioluminate 2.9 software by changing polar amino acids present in the active site to non-polar and vice-versa. The resultant change in stability of the mutants in comparison to wild-type protein was analyzed, and change in binding free-energy was calculated. Viable mutations from in silico analysis were selected to develop a series of affinity mutants using the site-directed mutagenesis kit (Stratagene, USA). A non-viable mutation with positive change in binding free energy was selected to create a GEII-control construct. Ligand titration curve was plotted for the developed mutants which was used to determine their dissociation constant and operating range.

### 2.6. Monitoring of Isoleucine in Living Bacterial Cells

For monitoring of isoleucine in bacterial cells, the *E. coli* BL21(DE3) containing pRSET-B-GEII were incubated for 48 h at 21 °C. For appropriate protein folding of nanosensor protein, the culture was stored in 4 °C overnight. The bacterial cells were centrifuged and the resultant pellet of the bacterial cells was resuspended in MOPS buffer (20 mM). Twenty microliter 10 mM isoleucine was added to 180 µL resuspended bacterial cell in the wells of microplate. The ratio of fluorescence emission intensities of Venus/ECFP was recorded for 45 min. Similarly, the emission was recorded with 10 mM each of leucine, valine, methionine, arginine, and cysteine.

### 2.7. Real-Time Monitoring of Isoleucine in Yeast Cells

The GEII in pYES-DEST vector was expressed in Saccharomyces cerevisiae/URA3 strain BY4742. The transformed yeast cells were grown on SD medium for three days after inducing 1% galactose along with 2% dextrose as a carbon source. The expression of ECFP-LivJ-Venus was controlled by the GAL1 promoter. Slides were treated with poly-L-lysine before imaging. Live imaging of yeast cells expressing GEII was carried out using confocal microscope (Leica DMRE) provided with confocal head TCS-SPE (Leica, Germany). A 63× oil immersion objective was used to measure the uptake of isoleucine by yeast cells after adding 10 mM of isoleucine on the slide. Ratio of emission intensity of Venus/ECFP was recorded for 350 s using LAS-AF software (Leica, Germany) without subtracting background fluorescence. Excitation filter was 436/20 nm. Emission filters were 480/40 nm and 535/35 nm for ECFP, and Venus, respectively.

## 3. Results and Discussion

A FRET-based isoleucine specific sensor, GEII, was constructed by flanking *E. coli* derived LivJ with ECFP and Venus at N- and C-terminus, respectively. Although, LivJ binds to all the three branched chain amino acids, it binds with the highest affinity to isoleucine (K_d_ with Ile = 0.9 μM, K_d_ with Leu = 2.3 μM, K_d_ with Val = 4 μM). The ECFP and EYFP (Enhanced Yellow Fluorescent Protein) have been commonly used as FRET pair in a number of studies [19,20]. The performance of this FRET pair was further improved by substituting EYFP with Venus; a variant of the EYFP. The Venus is a brighter, and faster folding Fluorescent protein, as compared to the EYFP and has reduced pH and halide sensitivity [23]. Faster maturation of the acceptor fluorophore, coupled with increased brightness, implies a more robust FRET signal. As a result, ECFP/Venus has been used in this study as a FRET pair. The Förster radius of the ECFP/mVenus FRET pair has been previously reported as 4.95 nm. Figure 1B shows a schematic representation of the sensor. The sensor was analyzed in silico, followed by in vitro characterization and in vivo imaging studies in bacterial and yeast cells.

### 3.1. In Silico Docking Studies

Before carrying out in vitro and in vivo experiments with GEII, in silico docking studies were carried out to better understand the molecular interactions between LivJ and the branched-chain amino acids. Molecular mechanics energy combined with the generalized Born and surface area continuum solvation (MM/GBSA) method was performed for calculating the approximate binding free energies [24]. The results showed that the maximum free energy change occurred for the interaction of LivJ with isoleucine (Table S1). The docking pose of isoleucine with respect to LivJ in the bound complex is shown in Figure 1C, while Figure 1D is a pictorial representation of the open and closed forms of LivJ in the absence, and presence of isoleucine, respectively. Figure 1E illustrates the interactions between LivJ residues and isoleucine as obtained from docking experiments. Isoleucine is bound to LivJ through hydrophobic interactions with Tyr202, Tyr150, Cys78, Ala101, Ala100, Ala103, Leu77, Phe276, Tyr18 and by polar interactions with Ser79 and Thr102.

### 3.2. Spectral Analysis of Nanosensor

**Figure 2:**
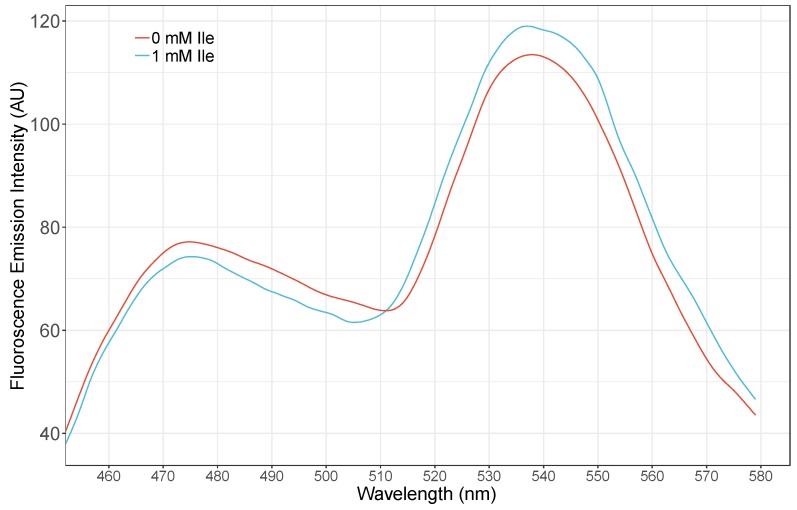
Fluorescence Spectral analysis of the GEII nanosensor. Change in the FRET ratio of the sensor was measured by using purified protein from bacterial cells in the absence and presence of 1 mM isoleucine. ECFP was excited at 435 nm and emissions from Venus recorded in the range of 480–535 nm.

**Figure 3:**
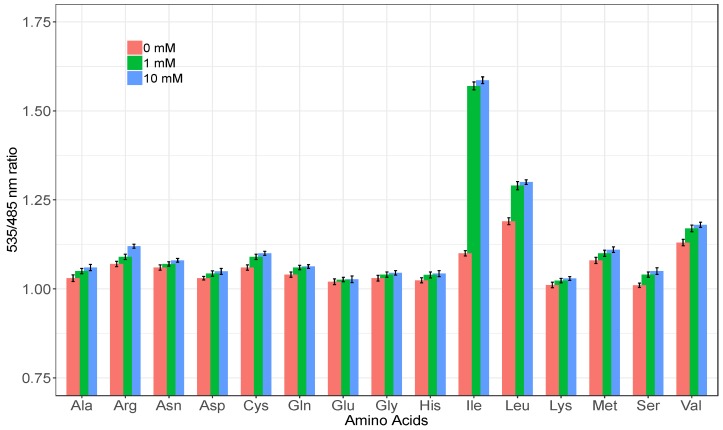
Specificity analysis of the GEII nanosensor. FRET ratio of the GEII sensor in absence and presence of 1 and 10 mM of different amino acids. All values are the mean of three independent experiments. Error bars indicate standard deviation. Excitation filter/slits (430/20 nm) and emission filters/slits for ECFP (485/20 nm) and Venus (535/25 nm) were used.

Spectral analysis of the GEII sensor revealed changes in the fluorescence emission spectra of the purified sensor protein on addition of 1 mM isoleucine. There was increase in the emission intensity of Venus and decrease in the emission intensity of ECFP, showing that FRET is occurring by the addition of isoleucine with the nanosensor protein (Figure 2). In addition, the GEII depicted a significant change in FRET ratio (535/485 nm fluorescence ratio) of over 44% from 1.1 to 1.6 (Δ_FRET-RATIO_ = 0.5) on exposure to 10 mM isoleucine (Figure 3). This permits GEII to reliably detect even small changes in isoleucine concentration under high noise conditions. It must be noted that such large dynamic ranges are relatively uncommon for PBP-based sensors, with a number of sensors exhibiting only moderate FRET ratio changes (Δ_FRET-RATIO_ < 0.3) [25,26,27]. The explanation of the impressive dynamic range of GEII is two-fold. Firstly, the N- and C-termini, to which the FRET fluorescent pairs are attached in GEII, lie in opposite domains of LivJ. Secondly, the magnitude of relative rigid body rotation between the two lobes in LivJ (50° and 60° from “open” and “super-open” states respectively to the “closed” state) is considerably large as compared to the typical conformational change on ligand binding seen in PBPs [28]. The combination of the above two factors ensures that the conformational change in LivJ on isoleucine binding directly translates to a large change in inter-fluorophore distance. This, in turn, results in a pronounced change in the FRET ratio of the GEII sensor. In contrast, additional engineering efforts are often required to achieve similar levels of FRET ratio change. For example, FRET sensors based on PBPs such as HisJ, AncQ, YbeJ, ArtJ, and GltI, harbor the N- and C- termini in the same lobe. To increase the change in the inter-fluorophore distance on ligand binding, a circular permutation of the periplasmic binding protein to place N- and C- termini, in opposite domains, is necessary [29]. Furthermore, for PBPs with smaller conformational changes on ligand binding as compared to LivJ, optimization techniques, such as usage of rigid linkers [30], linker truncation and insertion of fluorophores in the PBP [31] are required to increase the sensor’s signal-to-noise ratio.

### 3.3. Analysis of Specificity of the Nanosensor

Specificity analysis of the GEII nanosensor with different amino acids revealed that the change in FRET ratio was the maximum with isoleucine (Figure 3) and response to other amino acids i.e., valine, arginine, methionine, cysteine, alanine, asparagine, aspartic acid, glutamine, glutamic acid, glycine, histidine, lysine and serine, was minimal. In line with the observations in previous crystallographic studies [28], the sensor also responded to leucine, albeit only mildly, as compared to isoleucine. A plausible explanation for this observation is the existence of variations in conformations of the ligand-bound protein structure that are not detectable in X-ray crystallographic experiments, but can result in different FRET responses, nevertheless. Since the various states in the conformational ensemble may get merged in X-ray crystallographic experiments, a molecular dynamics simulation of the binding of isoleucine and leucine with the sensor protein may help elucidate the sensor response to these ligands. The GEII sensor was also tested for its response to the most common metal ions present in the human body, viz., Na^+^, K^+^, Ca^2+^, and Mg^2+^. The sensor did not undergo any significant change in FRET ratio (data not shown) for any of these metal ions. This showed that the sensor is not affected by the metals of cells.

### 3.4. Affinity of the GEII Nanosensor and its Mutants

**Figure 4:**
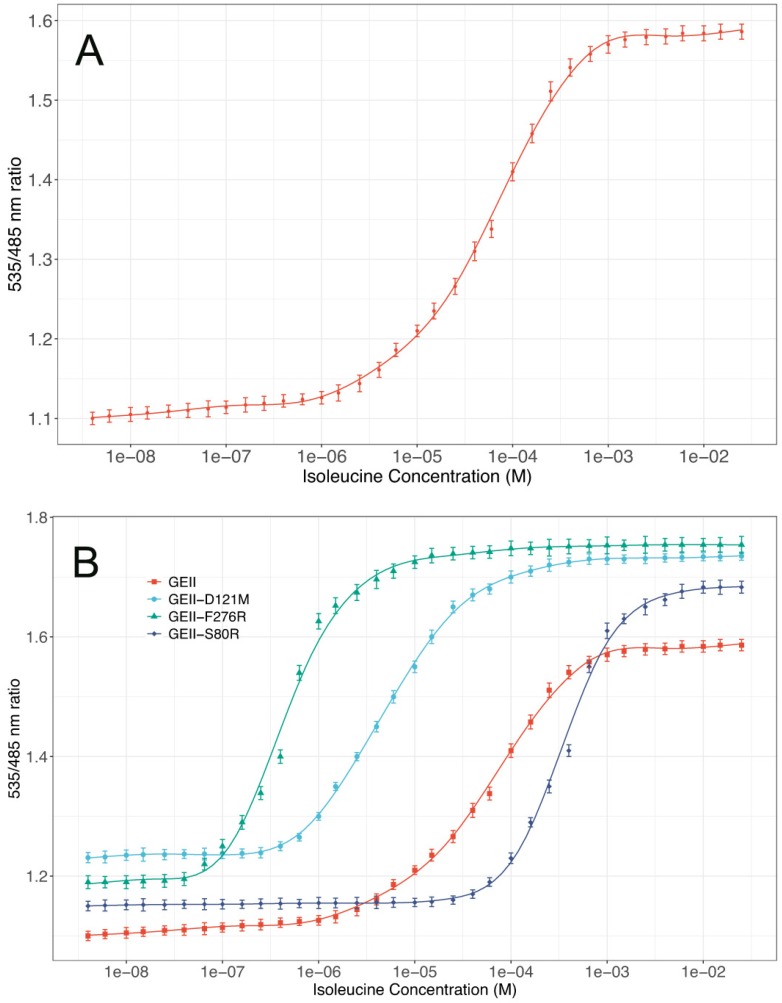
(**A**) Dissociation constant (K_d_) analysis of the GEII sensor in vitro by fitting the ligand titration curve at 300 K to a single binding isotherm equation. K_d_ equals the half-maximal saturation point on the sigmoidal curve. In this case, K_d_ equals 63 ± 6 μM. (**B**) Ligand binding isotherms for a library of affinity mutants of the GEII sensor. While, GEII-S80R showed reduced affinity relative to GEII, the other two mutants, GEII-F276R and GEII-D121M, had markedly improved affinities for isoleucine. The continuous lines correspond to the best fit curve for titration data obtained by non-linear regression using Generalized Additive Model (GAM) with cubic regression splines.

To calculate the operating range of the GEII sensor, a binding isotherm (Figure 4A) was plotted at 300 K by adding gradually increasing concentrations of isoleucine ranging from 4 nM to 25 mM (Table S2). The dissociation constant of the GEII sensor, corresponding to the half-maximal saturation point on the binding isotherm curve was determined to be 63 ± 6 μM and had an operating range of 3 μM to 382 μM, as calculated by the isoleucine concentration corresponding to 10% and 90% saturation of the sensor. Therefore, the sensor is suitable for tracking plasma isoleucine whose physiological concentration ranges from 45 to 105 μM [15,17].

Isoleucine concentration in mammalian cells is slightly higher and runs into a few hundred micro-molars. For example, in human lymphoblasts, its concentration ranges from 300–500 μM [32]. In mammalian batch cultures, its concentration varies from 770–1750 μM in BSC 24 cells, and from 700–1430 μM in TF 70 R cells [33]. Therefore, a library of affinity mutants was created to cater to the above use-case and to further expand the operating range of the sensor. To identify viable mutants, in silico random mutagenesis with Bioluminate software was carried out to predict the changes occurring in binding free energies of the protein-ligand interaction on mutating single residues. MM/GBSA ∆G_bind_ was found to be negative for three point-mutations: Ser to Arg mutation at position 80, Phe to Arg mutation at position 276 and Asp to Met mutation at position 121 (Table S3). For the rest of the mutations, change in binding free energy (∆G_bind_) was found to be positive indicating non-viable interactions. Therefore, one of these mutations: Tyr to Asp at position 202, was used to create a control construct. The control construct did not exhibit any change in FRET ratio on exposure to up to 100 mM isoleucine which suggests that it does not bind to isoleucine under experimental conditions as predicted by in silico analysis.

The three viable mutations were selected for experimental verification, and dissociation constants determined for each of the affinity mutants (Figure 4B). While GEII-S80R showed a reduced affinity for isoleucine vis-à-vis GEII (K_d_ = 411 ± 17 μM), the other two mutations resulted in sensors with improved binding affinities. The dissociation constants of GEII-F276R and GEII-D121M were determined to be 518 ± 18 nM, and 5.3 ± 0.5 μM, respectively. Considered together, GEII and its mutants have an operating range of 96 nM–1.5 mM, which makes them suitable for measuring isoleucine concentration over the entire spectrum of physiological ranges. For example, while GEII is an appropriate choice for measuring blood plasma isoleucine concentration (45 μM to 105 μM), GEII-S80R with a detection range of 73 μM–1493 μM is more suitable for monitoring isoleucine in human lymphoblasts cells (300 μM–500 μM), and TF 70 R cells (700 μM–1430 μM). Appropriate mutants should be selected based on the target isoleucine concentration. Table 1 summarizes the characteristics of the developed affinity mutants.

### 3.5. Analysis of pH Stability of the Nanosensor

**Figure 5:**
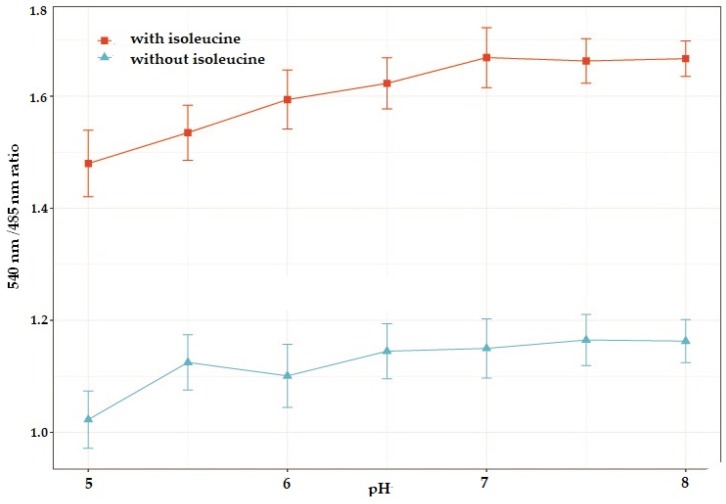
pH stability analysis of the GEII nanosensor. Measurement of the FRET ratio change of purified GEII nanosensor protein at different pH of the buffer solution in the absence, and presence of 10 mM isoleucine. Sensor protein concentration was kept at 0.20 mg/mL. All readings are averages of 3 independent experiment runs with the error bars indicating standard deviation.

To determine the suitability of the GEII sensor for physiological applications, the FRET response of the sensor was plotted against varying pH conditions, with 10 mM isoleucine and without isoleucine (Figure 5). As expected from sensors with ECFP-Venus FRET pair, GEII has a varying response to pH in the acidic range, which stabilizes in the basic range. Since pH is a highly controlled parameter in intracellular environments, the GEII sensor is appropriate for isoleucine measurements in physiological pH ranges. However, this also implies the non-applicability of the sensor to understand environments with acidic pH, such as vacuoles and secretory pathways. Development of FRET pairs immune to changes in pH can help surmount this limitation of the GEII sensor.

### 3.6. Measurement of Isoleucine in Live Bacterial Cells Using GEII Nanosensor

**Figure 6:**
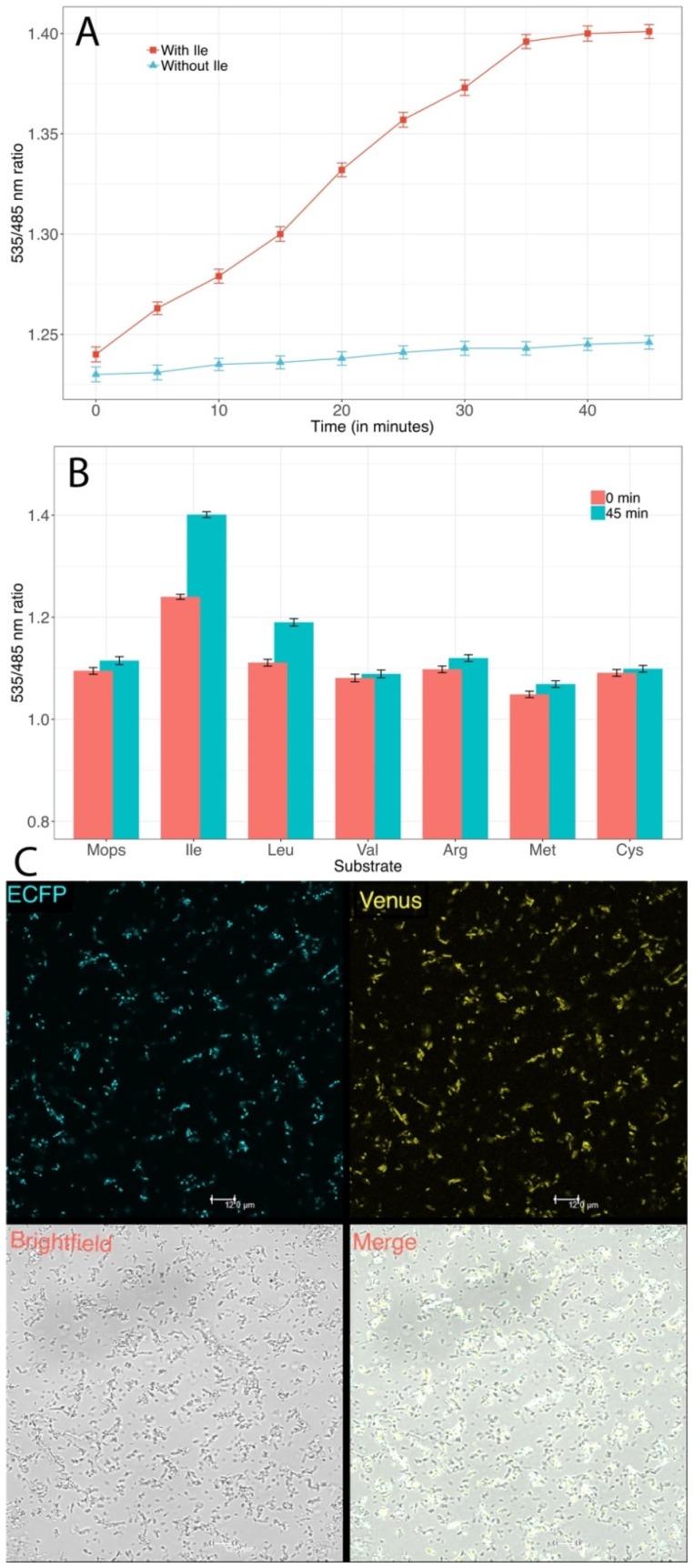
Real-time monitoring of isoleucine in bacterial cells. (**A**) Change in FRET ratio of the GEII sensor with respect to time when exposed to 10 mM isoleucine. The change is insignificant in the absence of isoleucine. Isoleucine uptake by bacterial cells took about 30–40 min to reach saturation levels. The sensor protein concentration of 0.20 mg/mL was used for the experiment. (**B**) FRET ratio change of expressed GEII sensor in *E. coli* cells after 45 min of exposure to 10 mM each of isoleucine, leucine, valine, methionine, arginine, and cysteine. Mops was used for the control experiment. As is clear from the maximum FRET ratio change with respect to isoleucine, GEII retains its specificity in bacterial cells as well. (**C**) Confocal images of the expressed GEII sensor in *E. coli* bacterial cells. Scale bar: 12 µM.

To quantify temporal dynamics of the sensor in bacterial cells, *E. coli* expressing the GEII sensor was exposed to 10 mM isoleucine and the FRET ratio tracked over time. Isoleucine uptake by bacterial cells took about 40 min to reach saturation levels manifesting as an increase in FRET ratio from 1.24 to 1.4 (Figure 6A). Another property of FRET sensors that must be verified in vivo, is their specificity which may be affected by the environmental sensitivity of the fluorescent pair in the altered environment. To confirm that the GEII sensor is specific to isoleucine in vivo, the *E. coli* cells expressing the GEII were exposed to 10 mM of arginine, cysteine, isoleucine, leucine, valine, and methionine each. In line with the in vitro observations, the GEII showed maximum FRET ratio change for isoleucine, followed by leucine (Figure 6B). Furthermore, real-time imaging of the *E. coli* expressing the GEII was done through confocal microscopy to visualize the cytosolic uptake of isoleucine in bacterial cells (Figure 6C). These results clearly indicate that the GEII sensor is robust enough for real-time monitoring of isoleucine in bacterial cells.

### 3.7. Live cell of Imaging of Isoleucine in Yeast Cells

**Figure 7:**
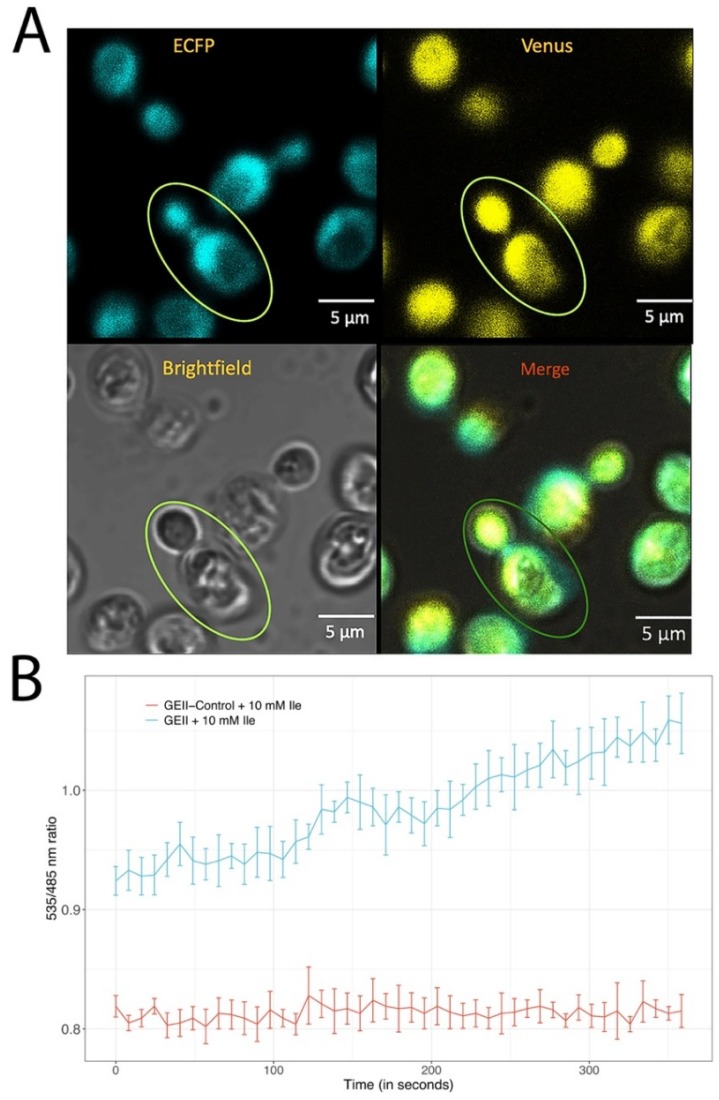
Live cell imaging of isoleucine in yeast cells. (**A**) GEII expression in yeast cells (*S. cerevisiae*) showing the confocal images; the selected region in green oval indicates the region of interest (ROI). (**B**) Ratiometric measurements from the GEII sensor in yeast cells when 10 mM of isoleucine was added to cells expressing GEII and GEII-control. Error bars indicate standard deviation in readings from three independent experiments. Scale bar: 5 µM.

To validate the utility of the GEII sensor in eukaryotic systems, it was expressed in *Saccharomyces cerevisiae* cells. Live cell imaging of the isoleucine using the GEII nanosensor was done using confocal microscopy, and the FRET ratio change at different time intervals was recorded for a duration of 350 s. A continuous increase in the FRET ratio was recorded from 0.92 to 1.05 after the addition of 10 mM isoleucine to the embedded yeast cells (Figure 7; Figure S3). The GEII sensor successfully detected the cytosolic uptake of isoleucine in yeast cells. Video recording of the yeast cells expressing the GEII was also done at different times (Video S1). The data showed that small amounts of isoleucine can be monitored only in few seconds through this nanosensor. The dark region in the images represents the vacuole, where the pH is too acidic to allow expression of the nanosensor. To exclude any artifacts, response of the control construct GEII-Control, which does not undergo any change in FRET ratio on exposure to 100 mM isoleucine, was also plotted. The results demonstrated the feasibility of the GEII sensor for real-time monitoring of isoleucine in eukaryotic system. Earlier nanosensors of different metabolites were also characterized in yeast through live cell imaging [34,35].

## 4. Conclusions

In this study, a robust isoleucine-specific genetically encoded sensor (GEII) was developed, which was used to measure isoleucine concentrations in vitro, and for live-cell imaging in bacterial and yeast cells. In addition, in silico mutagenesis, followed by experimental verification, was performed, in order to create a library of affinity mutants with a cumulative operating range of 96 nM–1.5 mM. To the best of our knowledge, GEII is the first isoleucine specific, non-invasive, genetically encoded nanosensor that can be used to measure concentrations at the cellular/sub-cellular level. Since GEII translates isoleucine concentration into fluorescence readouts, it can also be potentially used in High Throughput Screening (HTS) experiments to filter out bacterial strains with high isoleucine yield. Therefore, GEII would not only enable a better understanding of the role of isoleucine in various physiological processes, but it may also have commercial applications.

## Figures and Tables

**Table 1:** In vitro characterization of GEII and its affinity mutants.

Sensor	Mutation	K_d_ *	Operating Range ^†^	ΔR_max_^§^
GEII	Wild-Type	63 μM ± 6 μM	3 μM–382 μM	0.49
GEII-F276R	F276R	518 nM ± 18 nM	96 nM–4228 nM	0.56
GEII-D121M	D121M	5.3 μM ± 0.5 μM	0.8 μM–69 μM	0.53
GEII-S80R	S80R	411 μM ± 17 μM	73 μM–1493 μM	0.50
GEII-Control ^¶^	Y202D	>100 mM	Not defined	Not defined

* K_d_ determined from FRET binding isotherm curve (Figure 4B). ^†^ Range of isoleucine concentration in which the sensor can be used. The left and right ends of the operating range of the sensor correspond to the isoleucine concentration at which the sensor is 10% and 90% saturated, respectively. ^§^ Maximal change in the FRET ratio of the sensor. ΔR_max_ = R_max_ − R_min_, where R_max_ and R_min_ correspond to the maximum, and minimum FRET ratio in presence and absence of isoleucine, respectively. ^¶^ GEII-Control does not undergo any change in FRET ratio on exposure to up to 100 mM isoleucine, and therefore, can be used as a negative control.

## Supplementary Materials

The following are available online at https://www.mdpi.com/1424-8220/20/1/146/s1, Figure S1: Nucleotide sequence of the GEII construct, Figure S2: SDS-PAGE analysis of the purified sensor protein, Figure S3: Normalized data showing the changes in ECFP and Venus fluorescence intensity with time on exposure of yeast cells (*S. cerevisiae*) to 10 mM isoleucine. Table S1: Calculation of docking score and MM/GBSA (Molecular Mechanics/Generalized Born Surface Area) ΔG_bind_ using PDB-ID-1Z17, with respect to various amino acids, Table S2: Raw results for the titration experiments with GEII and its mutants, Table S3: In silico mutations in LivJ protein from polar to non-polar residues and vice-versa, Video S1: Live-monitoring of isoleucine uptake in yeast cells.
