# Supplementary material for: Real-Time Optical Detection of Isoleucine in Living Cells through a Genetically-Encoded Nanosensor
Source: Sensors (Basel). 2019 Dec 25;20(1):146. doi: 10.3390/s20010146 (PMC6983066; doi:10.3390/s20010146)
Supplement: Supplementary file 1 [file sensors-20-00146-s001.zip › supplementry/sensors-624368 -supplementry-for final.docx]

Supplementry

Real-Time Optical Detection of Isoleucine in Living Cells through a Genetically-Encoded Nanosensor

Shruti Singh ^1^, Maheshwar Prasad Sharma ^1^, Abdulaziz A. Alqarawi ^2^, Abeer Hashem ^3,4^, Elsayed Fathi Abd_Allah ^2^ and Altaf Ahmad ^5,^*

^1^ Department of Botany, School of Chemical and Life Sciences, Jamia Hamdard, New Delhi 110062, India; 189shrutis@gmail.com (S.S.); mpsharma@jamiahamdard.ac.in (M.P.S.)

^2^ Plant Production Department, College of Food and Agricultural Sciences, King Saud University, P.O. Box 2460, Riyadh 11451, Saudi Arabia; alqarawi@ksu.edu.sa (A.A.A.); eabdallah@ksu.edu.sa (E.F.A.)

^3^ Botany and Microbiology Department, College of Science, King Saud University, P.O. Box. 2460, Riyadh 11451, Saudi Arabia; habeer@ksu.edu.sa

^4^ Mycology and Plant Disease Survey Department, plant pathology Research Institute, ARC, Gaza 12511, Egypt

^5^ Department of Botany, Aligarh Muslim University, Aligarh 202002, India

***** Correspondence: aahmad.bo@amu.ac.in


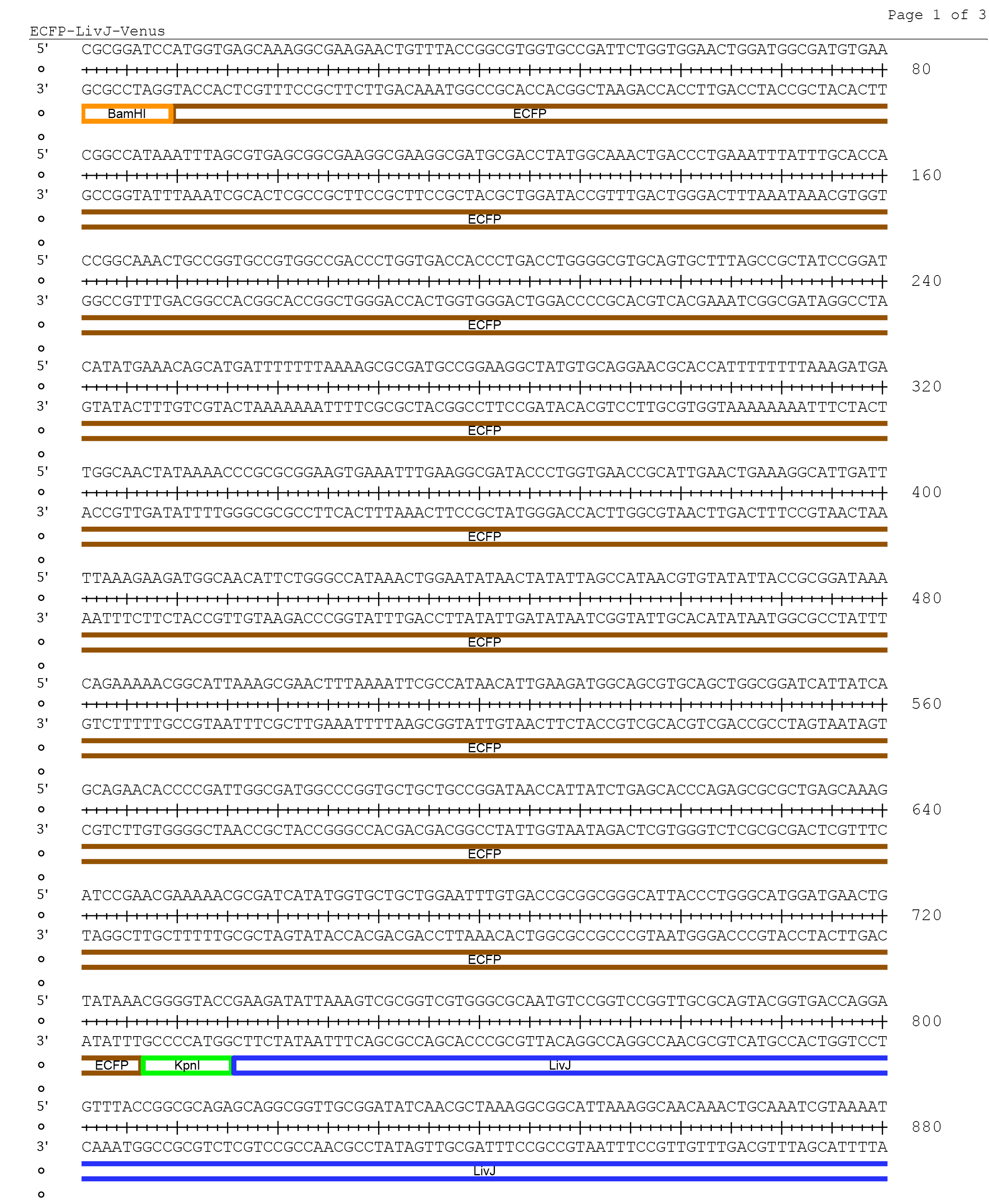


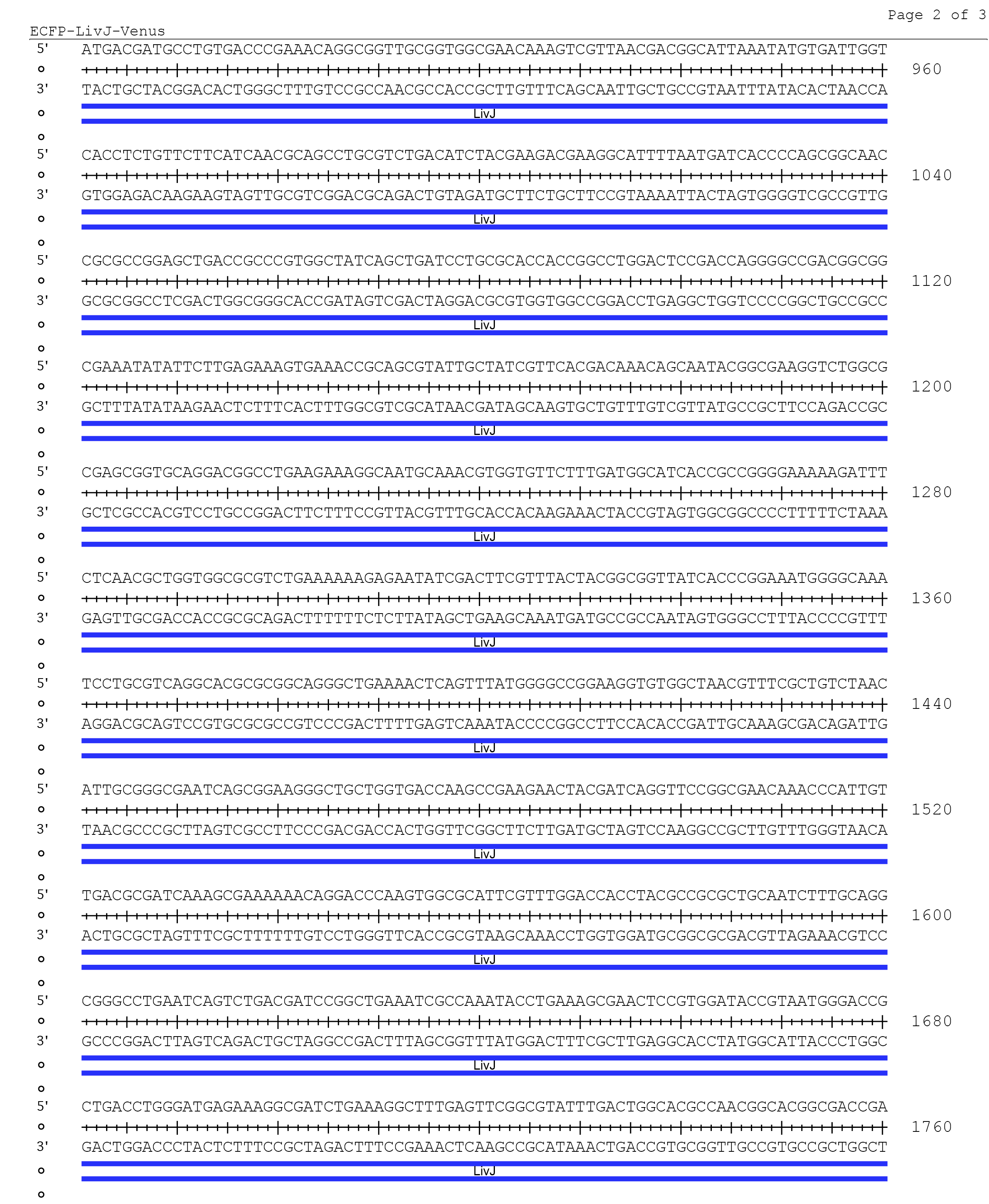


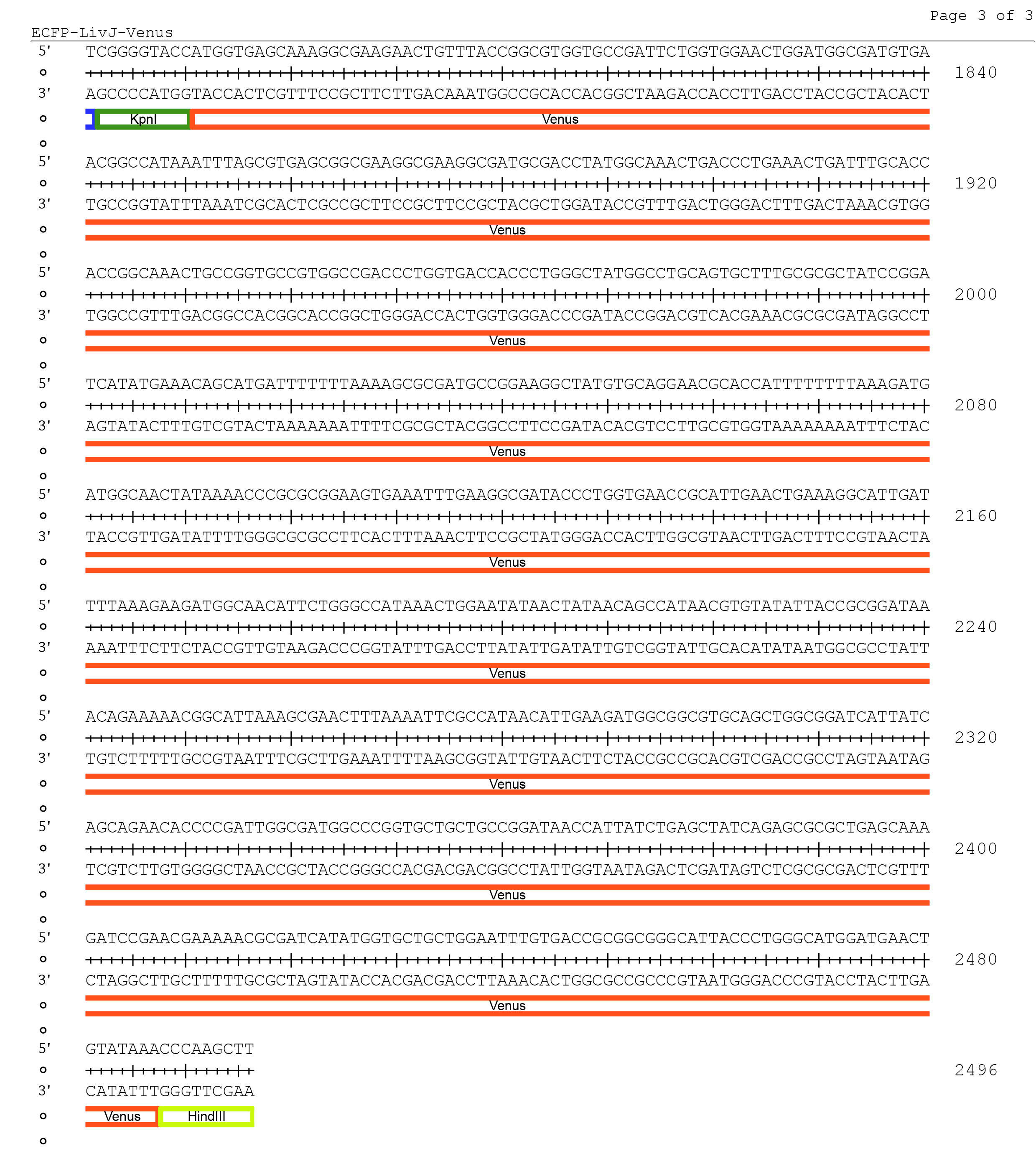


**Figure S1.** Nucleotide sequence of the GEII construct.


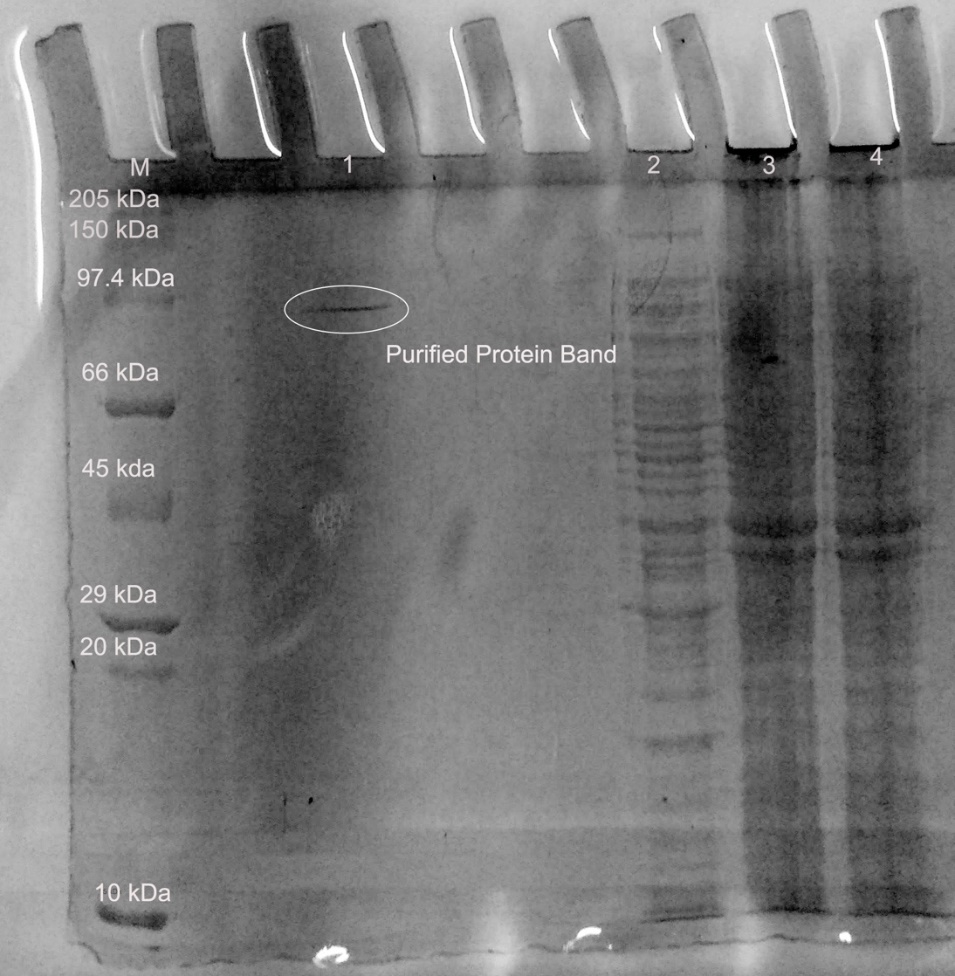


**Figure S2.** SDS-Page analysis of the purified sensor protein. M indicates marker, well 1: purified protein sample (2 μl), well 2: soup fraction (3 μl), well 3 and 4: pellet. The molecular weight of the sensor protein is 87.4 kDa.


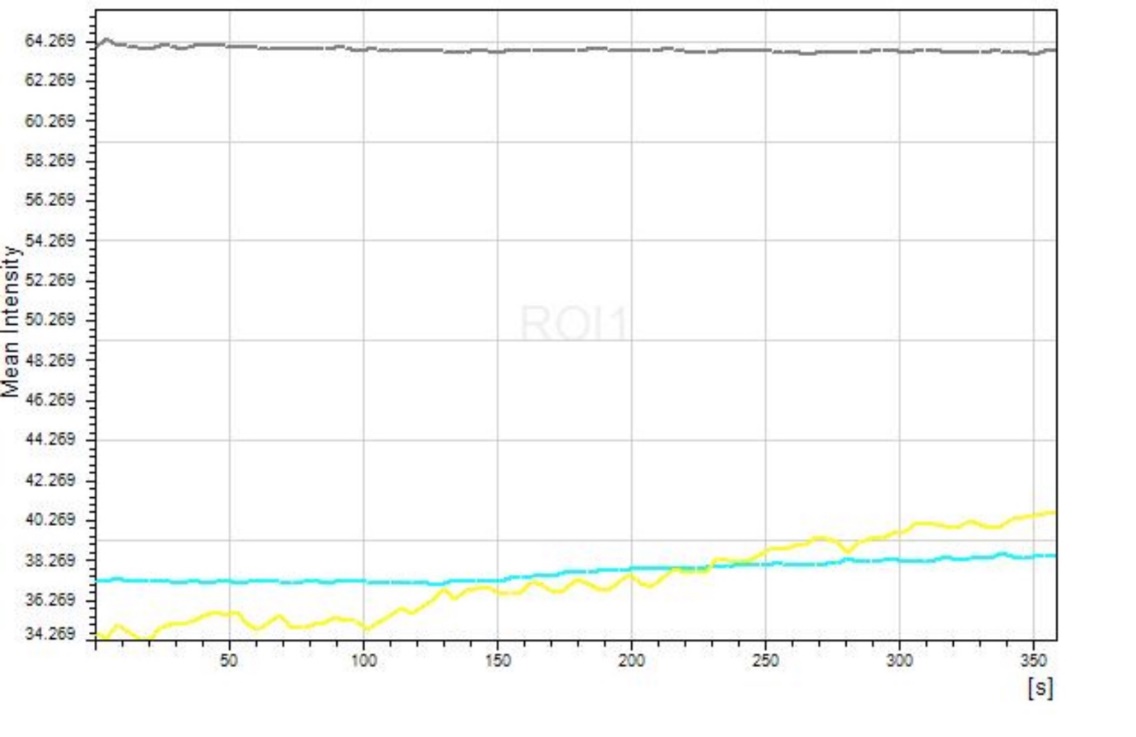


**Figure S3.** Normalized data showing the changes in ECFP and Venus fluorescence intensity with time on exposure of yeast cells (*S. cerevisiae*) to 10 mM isoleucine.

**Table S1.** Calculation of docking score and MM/GBSA ΔG_bind_ using PDB-ID-1Z17, with respect to various amino acids.

| **Amino Acid** | **Docking Score** | **MM/GBSA**  **ΔG_bind_** (**kJ/mol)** |
| --- | --- | --- |
| Isoleucine | −7.997 | −47.546 |
| Leucine | −7.665 | −41.8 |
| Valine | −7.552 | −42.188 |
| Threonine | −6.517 | −37.902 |
| Serine | −6.395 | −35.647 |
| Cysteine | −6.241 | −34.822 |
| Methionine | −5.834 | −28.376 |
| Alanine | −5.235 | −32.522 |
| Tyrosine | −4.868 | −20.13 |
| Cysteine | −3.674 | −6.076 |
| Glutamate | −1.607 | 23.054 |

**Table S2.** Raw results for the titration experiments with GEII and its mutants

| **Titation Curve for GEII with Ile** | | | **Titration Measurements for GEII-D112M** | | | | **Titration Measurements for GEII-S80R** | | | | | | **Titration Measurements for GEII-F276R** | | | | | | |  |  |
| --- | --- | --- | --- | --- | --- | --- | --- | --- | --- | --- | --- | --- | --- | --- | --- | --- | --- | --- | --- | --- | --- |
| Ile Conc. | Avg. FRET Ratio | SD | | Ile Conc. | Avg. FRET Ratio | SD | | Ile Conc. | Avg. FRET Ratio | | SD | | | Ile Conc. | | Avg. FRET Ratio | | SD | | |  |
| 4 nM | 1.100 | 0.0077 | | 4 nM | 1.231 | 0.0086 | | 4 nM | | 1.150 | | 0.0081 | | | 4 nM | | 1.190 | | 0.0107 | | |
| 6 nM | 1.103 | 0.0077 | | 6 nM | 1.232 | 0.0099 | | 6 nM | | 1.151 | | 0.0092 | | | 6 nM | | 1.190 | | 0.0095 | | |
| 10 nM | 1.000 | 0.0088 | | 10 nM | 1.235 | 0.0086 | | 10 nM | | 1.152 | | 0.0081 | | | 10 nM | | 1.190 | | 0.0107 | | |
| 15 nM | 1.107 | 0.0077 | | 15 nM | 1.236 | 0.0111 | | 15 nM | | 1.152 | | 0.0104 | | | 15 nM | | 1.191 | | 0.0107 | | |
| 25 nM | 1.109 | 0.0078 | | 25 nM | 1.236 | 0.0087 | | 25 nM | | 1.152 | | 0.0081 | | | 25 nM | | 1.192 | | 0.0107 | | |
| 40 nM | 1.110 | 0.0089 | | 40 nM | 1.237 | 0.0074 | | 40 nM | | 1.153 | | 0.0069 | | | 40 nM | | 1.195 | | 0.0108 | | |
| 65 nM | 1.112 | 0.0100 | | 65 nM | 1.237 | 0.0099 | | 65 nM | | 1.153 | | 0.0092 | | | 65 nM | | 1.220 | | 0.0098 | | |
| 100 nM | 1.114 | 0.0078 | | 100 nM | 1.238 | 0.0087 | | 100 nM | | 1.153 | | 0.0081 | | | 100 nM | | 1.250 | | 0.0113 | | |
| 160 nM | 1.117 | 0.0089 | | 160 nM | 1.238 | 0.0074 | | 160 nM | | 1.153 | | 0.0069 | | | 160 nM | | 1.290 | | 0.0116 | | |
| 250 nM | 1.119 | 0.0090 | | 250 nM | 1.239 | 0.0087 | | 250 nM | | 1.154 | | 0.0081 | | | 250 nM | | 1.339 | | 0.0107 | | |
| 400 nM | 1.122 | 0.0079 | | 400 nM | 1.250 | 0.0075 | | 400 nM | | 1.154 | | 0.0092 | | | 400 nM | | 1.403 | | 0.0112 | | |
| 630 nM | 1.124 | 0.0067 | | 630 nM | 1.265 | 0.0063 | | 630 nM | | 1.154 | | 0.0069 | | | 630 nM | | 1.540 | | 0.0123 | | |
| 1 µM | 1.126 | 0.0079 | | 1 µM | 1.300 | 0.0065 | | 1 µM | | 1.155 | | 0.0092 | | | 1 µM | | 1.626 | | 0.0130 | | |
| 1.5 µM | 1.132 | 0.0102 | | 1.5 µM | 1.350 | 0.0068 | | 1.5 µM | | 1.155 | | 0.0081 | | | 1.5 µM | | 1.652 | | 0.0132 | | |
| 2.5 µM | 1.144 | 0.0103 | | 2.5 µM | 1.398 | 0.0070 | | 2.5 µM | | 1.155 | | 0.0092 | | | 2.5 µM | | 1.674 | | 0.0134 | | |
| 4 µM | 1.161 | 0.0093 | | 4 µM | 1.449 | 0.0087 | | 4 µM | | 1.156 | | 0.0104 | | | 4 µM | | 1.696 | | 0.0153 | | |
| 6 µM | 1.186 | 0.0083 | | 6 µM | 1.501 | 0.0105 | | 6 µM | | 1.156 | | 0.0081 | | | 6 µM | | 1.710 | | 0.0120 | | |
| 10 µM | 1.210 | 0.0073 | | 10 µM | 1.550 | 0.0093 | | 10 µM | | 1.156 | | 0.0069 | | | 10 µM | | 1.725 | | 0.0104 | | |
| 15 µM | 1.235 | 0.0099 | | 15 µM | 1.600 | 0.0112 | | 15 µM | | 1.157 | | 0.0081 | | | 15 µM | | 1.736 | | 0.0122 | | |
| 25 µM | 1.266 | 0.0101 | | 25 µM | 1.650 | 0.0099 | | 25 µM | | 1.161 | | 0.0070 | | | 25 µM | | 1.739 | | 0.0104 | | |
| 40 µM | 1.310 | 0.0118 | | 40 µM | 1.670 | 0.0100 | | 40 µM | | 1.172 | | 0.0070 | | | 40 µM | | 1.741 | | 0.0104 | | |
| 60 µM | 1.338 | 0.0107 | | 60 µM | 1.680 | 0.0084 | | 60 µM | | 1.191 | | 0.0071 | | | 60 µM | | 1.742 | | 0.0105 | | |
| 100 µM | 1.410 | 0.0113 | | 100 µM | 1.700 | 0.0102 | | 100 µM | | 1.231 | | 0.0086 | | | 100 µM | | 1.748 | | 0.0122 | | |
| 160 µM | 1.458 | 0.0117 | | 160 µM | 1.710 | 0.0086 | | 160 µM | | 1.290 | | 0.0077 | | | 160 µM | | 1.748 | | 0.0105 | | |
| 250 µM | 1.511 | 0.0121 | | 250 µM | 1.720 | 0.0103 | | 250 µM | | 1.350 | | 0.0108 | | | 250 µM | | 1.749 | | 0.0140 | | |
| 400 µM | 1.541 | 0.0108 | | 400 µM | 1.725 | 0.0069 | | 400 µM | | 1.410 | | 0.0099 | | | 400 µM | | 1.751 | | 0.0123 | | |
| 650 µM | 1.558 | 0.0093 | | 650 µM | 1.730 | 0.0087 | | 650 µM | | 1.550 | | 0.0093 | | | 650 µM | | 1.752 | | 0.0105 | | |
| 1 mM | 1.570 | 0.0110 | | 1 mM | 1.730 | 0.0087 | | 1 mM | | 1.610 | | 0.0129 | | | 1 mM | | 1.753 | | 0.0140 | | |
| 1.5 mM | 1.576 | 0.0095 | | 1.5 mM | 1.730 | 0.0069 | | 1.5 mM | | 1.630 | | 0.0082 | | | 1.5 mM | | 1.753 | | 0.0088 | | |
| 2.5 mM | 1.579 | 0.0095 | | 2.5 mM | 1.731 | 0.0087 | | 2.5 mM | | 1.650 | | 0.0132 | | | 2.5 mM | | 1.754 | | 0.0140 | | |
| 4 mM | 1.580 | 0.0095 | | 4 mM | 1.732 | 0.0069 | | 4 mM | | 1.662 | | 0.0100 | | | 4 mM | | 1.754 | | 0.0105 | | |
| 6 mM | 1.584 | 0.0095 | | 6 mM | 1.733 | 0.0069 | | 6 mM | | 1.676 | | 0.0117 | | | 6 mM | | 1.754 | | 0.0123 | | |
| 10 mM | 1.584 | 0.0095 | | 10 mM | 1.734 | 0.0069 | | 10 mM | | 1.683 | | 0.0101 | | | 10 mM | | 1.754 | | 0.0105 | | |
| 15 mM | 1.586 | 0.0095 | | 15 mM | 1.734 | 0.0069 | | 15 mM | | 1.683 | | 0.0118 | | | 15 mM | | 1.754 | | 0.0123 | | |
| 25 mM | 1.586 | 0.0095 | | 25 mM | 1.735 | 0.0069 | | 25 mM | | 1.683 | | 0.0101 | | | 25 mM | | 1.754 | | 0.0140 | | |

**Table S3.** In silico mutations in LivJ protein from polar to non-polar residues and vice-versa. The three viable mutations with negative ΔG_bind_ and two sample non-viable mutations are shown below. Rest of the mutations, similar to the last two mutations, were found to be non-viable on account of positive ΔG_bind_.

| **Residue Position** | **Original Residue** | **Mutated Residue** | **Δstability (Solvated)** | **MM/GBSA**  **ΔG_bind_ kJ/mol** |
| --- | --- | --- | --- | --- |
| 80 | Ser | Arg | −11.21 | −5.494 |
| 276 | Phe | Arg | −23.96 | −13.572 |
| 121 | Asp | Met | −28.75 | −11.684 |
| 202 | Tyr | Asp | 40.77 | 9.282 |
| 276 | Phe | Asp | 35.57 | 1.687 |
